# Deciphering the Iron Side of Stroke: Neurodegeneration at the Crossroads Between Iron Dyshomeostasis, Excitotoxicity, and Ferroptosis

**DOI:** 10.3389/fnins.2019.00085

**Published:** 2019-02-19

**Authors:** Núria DeGregorio-Rocasolano, Octavi Martí-Sistac, Teresa Gasull

**Affiliations:** ^1^Cellular and Molecular Neurobiology Research Group, Department of Neurosciences, Germans Trias i Pujol Research Institute (IGTP), Badalona, Spain; ^2^Department of Cellular Biology, Physiology and Immunology, Universitat Autònoma de Barcelona, Bellaterra, Spain

**Keywords:** iron, reactive oxygen species, ferroptosis, stroke, excitotoxicity, iron dyshomeostasis, neurodegeneration, transferrin saturation

## Abstract

In general, iron represents a double-edged sword in metabolism in most tissues, especially in the brain. Although the high metabolic demands of brain cells require iron as a redox-active metal for ATP-producing enzymes, the brain is highly vulnerable to the devastating consequences of excessive iron-induced oxidative stress and, as recently found, to ferroptosis as well. The blood–brain barrier (BBB) protects the brain from fluctuations in systemic iron. Under pathological conditions, especially in acute brain pathologies such as stroke, the BBB is disrupted, and iron pools from the blood gain sudden access to the brain parenchyma, which is crucial in mediating stroke-induced neurodegeneration. Each brain cell type reacts with changes in their expression of proteins involved in iron uptake, efflux, storage, and mobilization to preserve its internal iron homeostasis, with specific organelles such as mitochondria showing specialized responses. However, during ischemia, neurons are challenged with excess extracellular glutamate in the presence of high levels of extracellular iron; this causes glutamate receptor overactivation that boosts neuronal iron uptake and a subsequent overproduction of membrane peroxides. This glutamate-driven neuronal death can be attenuated by iron-chelating compounds or free radical scavenger molecules. Moreover, vascular wall rupture in hemorrhagic stroke results in the accumulation and lysis of iron-rich red blood cells at the brain parenchyma and the subsequent presence of hemoglobin and heme iron at the extracellular milieu, thereby contributing to iron-induced lipid peroxidation and cell death. This review summarizes recent progresses made in understanding the ferroptosis component underlying both ischemic and hemorrhagic stroke subtypes.

## Iron Transport to the Brain: the Role of the Blood–Brain Barrier

Iron is essential for life; numerous proteins require iron as a cofactor for their activity. Iron acts as an electron donor or acceptor, and thus is pivotal for oxygen transport (coordinated with hemoglobin), cellular respiration (as a part of heme-containing cytochromes and proteins containing Fe–S groups in the electron transport chain), and DNA synthesis (in ribonuclease reductase). Moreover, in the nervous system, iron is essential for myelinization and neurotransmitter biosynthesis.

Iron exists both as Fe^2+^ and Fe^3+^; redox-active and cycling between these two states lead to the generation of reactive oxygen species (ROS) through the Fenton reaction. The ROS promote oxidative stress and cause extensive lipid peroxidation (Gutteridge et al., 1979). The brain is particularly vulnerable to lipid peroxidation damage because it is rich in polyunsaturated fatty acids (PUFAs) and iron, but relatively poor in antioxidant defenses (Cobley et al., 2018). The iron content remarkably differs across different brain regions: it is high in the striatum, medium in the hippocampus and cortex, and low in the pons and medulla (Ramos et al., 2014). Although it has recently attracted considerable attention, iron metabolism in the brain is yet not completely understood as that in other murine tissues (Ashraf et al., 2018). Brain imports iron from systemic circulation, and the levels of iron are determined by diet, intestinal iron absorption, release of iron initially stored in the hepatocytes, and export of iron recycled from macrophages. In addition, the levels of iron in the plasma are regulated by the hormone hepcidin through its effect on the transmembrane iron export protein ferroportin (FPN), which is mainly present at the cell membrane of macrophages, hepatocytes, and enterocytes.

In the blood and most other extracellular fluids, iron is essentially carried bound with high affinity to the iron transport protein transferrin (Tf), which shields Fe^3+^ from redox activity. In addition to Tf, iron also associates with other carrier proteins in the blood such as ferritin (FT), haptoglobin (Hp), Hpx, or albumin. Recently, half a dozen different non-proteinaceous low-molecular-weight iron species (<10 KDa) have been shown to exist in the blood (Dziuba and Lindahl, 2018); further studies are required to determine the precise identity and physiological role of these species. Once iron is bound to Tf, Fe^3+^ is rendered redox-inactive and virtually non-exchangeable or displaceable by other physiological metals or molecules. Tf-iron complexes circulate in the blood until they bind transferrin receptors (TfRs) in cellular membranes of target cells. In general, in non-iron-overload conditions, little, if any, non-transferrin-bound iron (NTBI) is found in the blood of healthy individuals (Gaasch et al., 2007). The high affinity of Tf for iron and the large amount of Tf in the blood exceed the total requirement for iron binding; in normal human blood, the saturation of transferrin with iron (TSAT) is only of around 30% (De Valk et al., 2000; Van Dijk et al., 2008). Therefore, iron transport to the brain is mainly from iron-loaded transferrin (holotransferrin, HTf) through the endothelial cells of the BBB and the epithelium of the choroid plexuses and arachnoid membrane, the blood–cerebrospinal fluid (CSF) barrier.

**FIGURE 1:**
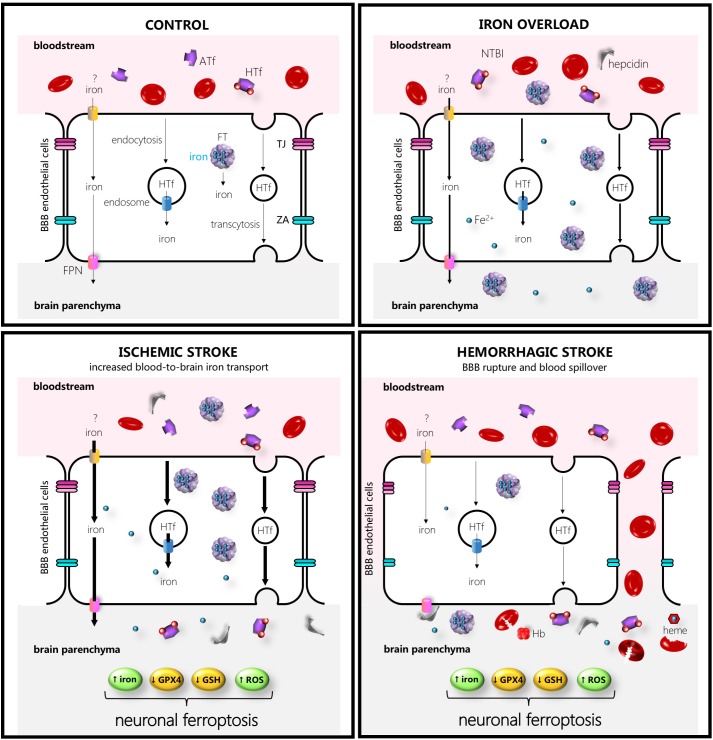
Schematic drawing of some of the main players in iron transport to the brain in control (left hand upper side), iron overload (right-hand upper side), ischemic stroke (left-hand lower side), or intracerebral hemorrhage (right-hand lower side). The thicker the arrows the more increased the transport. Abbreviations: ATf, apotransferrin; FPN, ferroportin; HTf, holotransferrin; FT, ferritin, GPX4, glutathione peroxidase 4; GSH, glutathione; Hb, hemoglobin; NTBI, non-transferrin-bound iron; ROS, reactive oxygen species; TJ, tight junction; ZA, zonula occludens.

**FIGURE 2:**
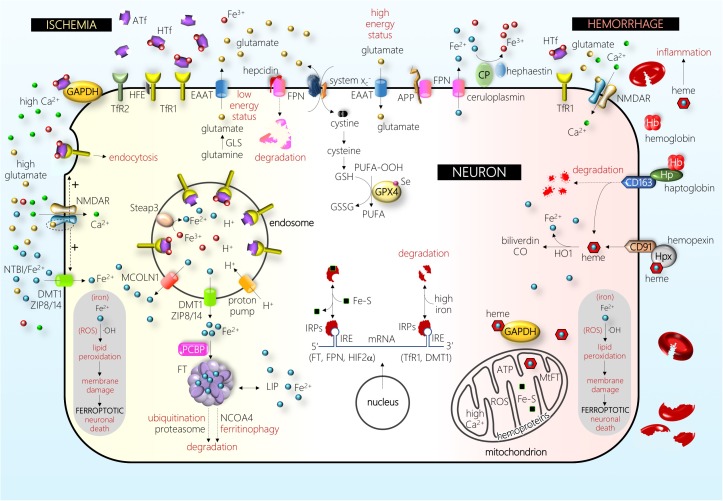
Schematic drawing of some of the main players in a neuron under ischemic (left-hand side) and hemorrhagic (right-hand side) stress (see text for details). Abbreviations: APP, amyloid precursor protein; ATf, apotransferrin; CD163, hemoglobin/haptoglobin receptor; CD91, heme/hemopexin receptor; CO, carbon monoxide; CP, ceruloplasmin; DMT1, divalent metal transporter 1; EAAT, excitatory amino acid transporter; Fe^2+^, ferrous iron; Fe^3+^, ferric iron; Fe–S, iron–sulfur cluster; FPN, ferroportin; FT, ferritin; GAPDH, glyceraldehyde-3-phosphate dehydrogenase; GLS, glutaminase; GPX4, glutathione peroxidase 4; GSH, glutathione; GSSG, glutathione disulfide; Hb, hemoglobin; HFE, hereditary hemochromatosis protein; HIF, hypoxia-inducible factor; HO1, heme oxygenase 1; Hp, haptoglobin; Hpx, hemopexin; HTf, holotransferrin; IRE, iron-responsive element; IRPs, iron regulatory proteins; LIP, labile iron pool; MCOLN1, mucolipin 1; MtFT, mitochondrial ferritin; NCOA, nuclear receptor coactivator; NMDAR, NMDA receptor; NTBI, non-transferrin-bound iron; OH, hydroxyl radical; PCBP, poly(rC)-binding protein; PUFA, poly-unsaturated fatty acid; PUFA-OOH, PUFA peroxide; ROS, reactive oxygen species; Se, selenium; Steap, six-transmembrane epithelial antigen of prostate; system $X_{c}^{-}$, cystine–glutamate antiporter; TfR1, transferrin receptor 1; TfR2, transferrin receptor 2; ZIP8/14, zinc transporters 8/14. The picture depicts the effect of one of the major players in the pathophysiology of brain I, the disruption of glutamatergic neurotransmission homeostasis that produces elevated extracellular glutamate levels, overactivation of the NMDA subtype of glutamate receptors (NMDAR), and excitotoxic cell death. In the recent past, different authors have shown that this NMDAR overactivation boosts neuronal iron uptake and produces an iron-dependent form of neuronal death associated with massive lipid peroxidation that fits with the definition of ferroptosis. Further, in the hemorrhage type of stroke, iron derived from hemoglobin and/or heme enters neurons and produces massive lipid peroxidation. Damage by ischemic and hemorrhagic stroke is alleviated by the inhibitors of ferroptosis.

Although barrier cells of the brain form tight junctions laterally, epithelial cells of the choroid plexuses are fenestrated and more permeable than the endothelial cells of the BBB (Strazielle and Ghersi-Egea, 2016). The abluminal side of brain capillary endothelial cells is surrounded by pericytes and astrocytes of the neurovascular unit that provide extra structural and functional support. TfRs are abundantly expressed in the luminal side of membranes of polarized endothelial cells; once they bind Tf, the TfR–Tf complexes undergo endocytosis. The following steps are still controversial, and different pathways are thought to be involved in brain iron acquisition (Bradbury, 1997; Khan et al., 2018). Thus far, the main contribution to this process is thought to include the following pathways: (1) endocytic vesicles containing TfR–HTf traveling from the luminal membrane to fuse to the abluminal membrane and exocytose either HTf or free iron into the brain extracellular space; (2) under low pH conditions within the endosome of endothelial cells, as in most cell types, iron is extracted from Tf, converted to Fe^2+^ by endosomal ferrireductases such as the six-transmembrane epithelial antigen of prostate protein 3 (Steap3), and released to the cytosol through divalent metal transporter 1 (DMT1) or the ion channels TRPML, also known as mucolipin 1 or MCOLN1 (detailed information can be found in references Luck and Mason, 2012; Skjorringe et al., 2015). Subsequently, Fe^2+^ from the cytosol is exported through FPN present in the abluminal membrane of endothelial cells, which are in contact with astrocytes expressing ceruloplasmin, that rapidly oxidize Fe^2+^ to Fe^3+^ (this Tf/TfR endocytosis mechanism is common for endothelial and neuronal cells and is depicted in Figure 1, 2). A recent study performed using comprehensive mathematical models concluded that optimal iron homeostasis is dependent on the degree of saturation and the transport of HTf across the BBB from the blood (Khan et al., 2018).

In addition, in mice exposed to experimental stroke under normal iron conditions, iron deposits in the microvasculature were observed early in the border zone of the damaged areas (Desestret et al., 2009), suggesting that, when signaled, the BBB might function as an iron reservoir from which iron is released into the brain. Regarding signaling to release, in bovine retinal endothelial cells, the FPN-mediated iron export is known to be blocked by the FPN inhibitor hepcidin (Hadziahmetovic et al., 2011). In addition, new insights into the communication between systemic iron status and brain iron uptake reveal the regulation of brain iron uptake at the level of brain microvascular endothelial cells rather than the BBB acting merely as a retaining wall (Chiou et al., 2018b). In support of this regulatory hypothesis, endothelial microvascular cell distribution of TfR is altered in luminal, intracellular, and abluminal membranes depending on brain iron status (Simpson et al., 2015), and microvascular cell iron availability and efflux have been found to be tightly modulated by diffusible ceruloplasmin and hepcidin released from astrocytes surrounding the BBB (McCarthy and Kosman, 2014). Moreover, a recent study showed that inhibition of DMT1 alters the transport of iron and Tf across the endothelial cells and proposed that the levels of iron-devoid Tf (apotransferrin) and iron delivered to the brain are strongly regulated by the ratio apotransferrin:HTf as well as by hepcidin levels in the extracellular fluid of each brain region (Duck et al., 2017).

Further, very recently, a new function for FT heavy chain (FTH) as a transporter of NTBI across the BBB by binding to the T-cell immunoglobulin and mucin domain-containing 1 receptor 1 (TIM-1) has been reported in human endothelial cells (Chiou et al., 2018b).

In the context of physiological iron-overload conditions found, for instance, in individuals on iron-rich diets, a significant pool of NTBI is found in the blood and might be readily absorbed by microvascular endothelial cells. This effect might be relevant considering that regional differences in the brain uptake of ^59^Fe–NTBI versus ^59^Fe–TfR have been reported (Deane et al., 2004), and that ^59^Fe–NTBI seems to be transported faster than ^59^Fe–TfR into the brain parenchyma of iron-overloaded disease-free mice. Furthermore, the iron storage protein FT has been found to be upregulated in epithelial cells of the choroid plexus and endothelial cells of the brain during systemic iron overload (Tripathi et al., 2017).

## Iron Uptake and Handling by Parenchymal Brain Cells

Once in the brain interstitial fluid, both TBI and NTBI are potential iron sources for brain cells (Gaasch et al., 2007); unlike blood Tf, CSF Tf is considered to be completely saturated with iron (Bradbury, 1997). Within the extracellular milieu of the brain parenchyma, iron is carried loaded into Tf or bound to low-molecular-weight molecules such as citrate, ascorbate, or ATP. Each brain cell type uptakes extracellular TBI or NTBI depending on the iron-transport receptors and channels present in their membranes and, physiologically, safely handles iron by using the network of proteins they specifically express to import, store, traffic, and export iron. Players of cellular iron homeostasis are cell type-specific, and the list of proteins involved in iron homeostasis is still growing. As an example of iron-related new proteins, only 3 years ago, the members of the ZIP family of metal ion transporters Zip8 and Zip14, which, unlike the DMT1 transporter are only active at normal physiological pH, were reported to be expressed by hippocampal neurons, and Zip8 was found to play a major role in the accumulation of NTBI by hippocampal neurons (Ji and Kosman, 2015), whereas both Zip8 and Zip14 were considered to play a role in iron homeostasis in rat retina (Sterling et al., 2017).

### Receptors Involved in Iron Uptake by Brain Cells

In addition to the well-known TfR1 as a main player of the TfR/Tf endocytic pathway in most brain cells, other proteins, e.g., transferrin receptor 2 (TfR2) (Kawabata, 2018) or glyceraldehyde-3-phosphate dehydrogenase (GAPDH) (Raje et al., 2007), have been described as receptors for Tf and are currently being investigated. TfR1 binds HTf with a Kd of 1 nM; it is expressed in response to changes in iron levels through modulation by iron regulatory elements (IREs) present in its mRNA and binds hereditary hemochromatosis HFE protein, a molecule that competes with HTf for binding to TfR1. In contrast, TfR2 has lower affinity for HTf; its levels are not modulated by IREs (West et al., 2000) and, although still controversial, seems to bind HFE protein in a sequence non-overlapping to that of the Tf-binding site (for a review see Worthen and Enns, 2014).

The TfR2 is present in two main isoforms, TfR2α and TfR2β. The TfR2α form has been reported to be more abundant in the brain, especially in the hippocampus; brain TfR2α levels were reported to significantly differ across animals fed iron-deficient or iron-enriched diets (Pellegrino et al., 2016). A specific role for TfR2 in the control of the iron regulatory network in the brain tissue has been recently suggested, as altered brain IRPs, iron accumulation, and reactive microglia signaling were found in TfR2-KO mice (Pellegrino et al., 2016). In contrast, a previous study on TfR2 mutant mice showed changes in hepatic, but not brain iron levels, although that study reported a 30% increase in brain FT levels (Acikyol et al., 2013).

In addition to TfR1 and TfR2, the multifunctional molecule GAPDH has been reported to work as a TfR in different cell types (Raje et al., 2007; Kumar et al., 2012) with a role in iron uptake and/or iron export (Sheokand et al., 2016) and as a chaperone for intracellular heme traffic (Sweeny et al., 2018). Relevant to brain and excitotoxicity, GAPDH interacts with the α-amino-3-hydroxy-5-methyl-4-isoxazolepropionic acid subtype of glutamate receptors in the neurons without glutamate stimulation and has also been reported to be pivotal for axon development (Lee et al., 2016).

Another receptor-mediated means to import iron into brain cells, at least in oligodendrocytes, is the uptake of FTH through TIM-1/2 receptors (Todorich et al., 2008, 2011; Chiou et al., 2018a). In other non-neuronal cells, FTH internalize through TfR1 (Li et al., 2010), whereas scavenger receptor class A member 5 (SCARA5) has been found to play also a role as a receptor for FT light chain uptake (Li et al., 2009). To the best of our knowledge, the role of SCARA5 has not yet been investigated in any brain cell type.

### Mechanisms and Proteins Involved in Cytosolic Iron Handling in Different Brain Cell Types

Inside the cell cytosolic and nuclear compartments, iron chaperone poly(rC)-binding proteins serve the binding and delivery of iron to iron-requiring enzymes, ubiquitous protein of iron storage FT, or the iron export membrane protein FPN (Philpott et al., 2017). In the cytosol, unused iron is stored into FT, a molecule composed of 24 subunits of a variable proportion of heavy and light chains, that shields up to 4500 iron atoms in a redox inert state. Neurons express both TfR1 and DMT1 (Pelizzoni et al., 2012; DeGregorio-Rocasolano et al., 2018); DMT1 is mainly found in the cytoplasm colocalizing with endosomes/lysosomes. Further, neurons express the iron export molecule FPN (Burdo et al., 2001; Zhou et al., 2017). Astrocytes express TfR1, DMT1, and FPN (Yang et al., 2012; Huang et al., 2014). Hence, although TfR1 is considered to be the main gate for cellular iron import, control astrocytes show low TfR1 levels, and most mature oligodendrocytes or microglial cells do not express TfR1 (Kaur and Ling, 1995; Rathnasamy et al., 2016). However, under hypoxia, both astrocytes and oligodendrocytes are found to upregulate TfR (Yang et al., 2012; Rathnasamy et al., 2016). Moreover, oligodendrocytes and cells of choroid plexuses, but not other brain cell types, express Tf under normal condition (Leitner and Connor, 2012). All the above are examples of the important heterogeneity in the expression and induction of iron homeostatic or IRPs observed in different brain cell types and situations. Most of the proteins involved in iron metabolism, including cytosolic FT levels, are mainly regulated by iron, oxidative stress, and inflammation. The iron-dependent regulation occurs mainly at the post-transcriptional level via the IREs and iron regulatory protein (IRP) machinery. When iron levels are high, iron binds to the IRP, which then loses affinity for the IRE sequence in FT mRNA, resulting in increased FT expression (the cytosolic iron handling is depicted in Figure 2) (Arosio et al., 2017). Several studies have shown that the mobilization of iron from FT requires FT degradation either by lysosome–autophagy or ubiquitin proteasome pathways (Asano et al., 2011; Mancias et al., 2015). In addition to its role in storing iron within the cell, FT can be secreted (Truman-Rosentsvit et al., 2018).

In addition to post-transcriptional regulation, transcriptional regulation has been reported for some of the proteins involved in iron homeostasis through the hypoxia-inducible factor family of transcription factors or the heme-dependent transcription factors (e.g., for TfR1 or FPN) (Marro et al., 2010; Man-man et al., 2017). Moreover, the master regulator of bodily iron homeostasis hepcidin is subjected to a sophisticated transcriptional regulation through the bone morphogenetic protein/SMAD pathway, interleukin 6 pathway, or erythropoietin/erythroferrone pathways when regulated according to the iron, inflammatory, or erythropoietic demands (Sangkhae and Nemeth, 2017). The regulation of cellular iron balance has been recently reviewed (Recalcati et al., 2017).

Iron also plays an important role in specific organelles; this is especially true for mitochondria. Mitochondria house the assembly of heme, the majority of the Fe–S cluster biosynthesis apparatus, and contain the respiratory complexes that need iron-containing cofactors. In addition, mitochondria are a major source of endogenous ROS. Sustained iron exposure increased mitochondrial ROS levels in dopaminergic neuroblastoma SH-SY5Y cells (Huang et al., 2017), and scavenging of mitochondrial ROS was protective against iron overload damage in hippocampal neurons, preserving mitochondrial morphological integrity and membrane potential (Pelizzoni et al., 2011). In addition, a recent study showed that sequestration of iron by mitochondrial FT was neuroprotective against oxidative stress and played an important role in preventing neuronal damage in some other conditions (You et al., 2016; Guan et al., 2017). Moreover, mitochondrial FT overexpression significantly reduced cellular labile iron pool, prevented H_2_O_2_-induced elevation of labile iron, and rescued cells from H_2_O_2_-induced damage (Gao et al., 2017).

In several cell types, although not yet studied in neurons, mitochondria-endosome interactions have been observed, suggesting a possible direct transfer of iron from endosomes to mitochondria (Das et al., 2016; Hamdi et al., 2016) through the “kiss-and-run” hypothesis.

## Iron Dyshomeostasis in the Brain

Brain iron abnormalities are associated to rare, but severe, neurodegenerative conditions, and there is growing evidence that the more common systemic iron overload disorders such as hereditary hemochromatosis exert important effects on the brain iron content and pathological brain iron deposition (Nandar and Connor, 2011; Kumar et al., 2016). Moreover, the hemochromatosis H63D polymorphism of the *HFE* gene could be a disease-modifying gene in frontotemporal lobar degeneration, fostering iron deposition in the basal ganglia (Gazzina et al., 2016), or even macro- and microanatomically altering some brain structures associated with changes in Tf levels in the blood (Jahanshad et al., 2012). Recently, abnormal recycling of TfR1 due to reduced post-translational palmitoylation has been reported to be crucial to affect the iron import in the so-called neurodegeneration with brain iron accumulation disease (Drecourt et al., 2018). Further, neurodegeneration with brain iron accumulation disease-linked genes that showed altered expression in response to iron loading has been recently reported to be directly or indirectly related to myelin metabolism (Heidari et al., 2016).

A list of mild conditions, including aging (Ward et al., 2014), continuous uptake of some bioavailable iron sources (Peters et al., 2018), or obesity (Han et al., 2017), have been reported to alter brain iron content and/or distribution. In addition, a recent report showed that iron administration increases iron levels in the brains of healthy rats, which induces brain changes and triggers a hormetic response that reduces oxidative damage (Piloni et al., 2018). Moreover, other studies reveal a complex interplay between inflammation and brain iron homeostasis (Righy et al., 2018; Sankar et al., 2018), with acute inflammation increasing non-Tf iron uptake by brain microglia (McCarthy et al., 2018). This dyshomeostasis is especially important in acute pathologies such as stroke, in which the impairment of the BBB regulatory role is rapidly and massively affected following either rupture of an artery (ICH) or aberrant increase of brain microvascular endothelial permeability following ischemic stroke (AIS). Dysregulation of iron resulting in the accumulation of free iron and brain iron overload, which can increase the production of ROS, is evident in aging and stroke-related pathologies and is also a hallmark of chronic and long-term neurodegenerative pathologies. In this regard, iron overload in the brain has been reported in Huntington, Parkinson, or Alzheimer diseases [readers interested in the topic are referred to recent detailed reviews by Morris et al. (2018) and Uranga and Salvador (2018)]. Interestingly, marked age-related changes in brain iron homeostasis have also been observed in amyloid precursor protein (APP)-knockout mice (Belaidi et al., 2018).

## Iron Overload Worsens Neurological Damage Both in Ischemic Stroke and Intracerebral Hemorrhage

Stroke is a life-threatening disease that causes high rates of permanent disability subsequent to neuronal loss. There are two major types of strokes: ischemic stroke or AIS, which is caused by a blood clot blocking an artery and accounts for 85% of all strokes, and hemorrhagic stroke or ICH, caused by leakage or rupture of an artery and accounts for 15% of stroke cases.

### Iron Overload Condition in Stroke Damage and Outcome

In ischemic stroke, neurons die because of interrelated processes that include glutamate excitotoxicity, excess of free radical production, and inflammation (Nagy and Nardai, 2017). Each of these mechanisms has already been targeted in clinical trials, although unsuccessfully (for a review see Chamorro et al., 2016). Moreover, free radical-induced oxidative stress has been for long recognized as a pathogenic factor in the ischemia/reperfusion (I/R) injury that follows reoxygenation. The hydroxyl radical, the most harmful of the free radicals, is generated through reactions catalyzed by iron (Kell, 2009; Chen H. et al., 2011). Of relevance to stroke damage and final outcome, several clinical studies indicate that a previous systemic iron overload condition, measured as high levels of serum FT at admission, is associated with increased brain damage and worse outcome induced by ischemic stroke (Davalos et al., 1994; Millan et al., 2007). A similar detrimental effect (increased ischemic damage) was observed in iron-overloaded animals exposed to experimental ischemic stroke (Castellanos et al., 2002; Gamez et al., 2003; Mehta et al., 2004; García-Yébenes et al., 2012; DeGregorio-Rocasolano et al., 2018). Iron overload is a major source of oxidative stress in ischemic brains (Carbonell and Rama, 2007). In addition, iron overload exacerbated the risk of hemorrhagic transformation in animal models of transient ischemia plus early reperfusion (García-Yébenes et al., 2012; García-Yébenes et al., 2018); therefore, in iron-overloaded animals, an initial ischemic event has higher probability to become an ICH. The contribution of iron overload to widespread the damage in the ICH is supported by the fact that systemic iron overload (estimated using high serum FT levels) was associated with higher perihematoma edema and poor outcomes in patients with ICH (Mehdiratta et al., 2008; Perez de la Ossa et al., 2010; Bakhshayesh et al., 2014; Garton et al., 2017). Further, a combination of high serum FT and low serum iron and Tf was associated with poor outcome in ICH patients (Yang et al., 2016). Therefore, systemic iron overload impacts the outcome of both ischemic and hemorrhagic stroke patients.

The brain is physiologically sheltered from fluctuations in systemic iron because of the BBB, even under experimentally induced diet iron overload conditions or intraperitoneal iron administration (Castellanos et al., 2002; Millerot et al., 2005). However, in the ischemic brain, the detrimental effect of iron overload might result from systemic iron pools reaching the brain parenchyma. Following artery occlusion, a physiological increase in transport through a still somehow intact BBB is noted, and thereafter pathological halt of the BBB function that results in leaky or broken capillaries that allow blood spill out (Figure 1). Some BBB impairment with aberrant exchange of brain and blood molecules occurs relatively early following ischemic stroke (within the first hours of post-symptom onset) showing a biphasic behavior in some studies (Klohs et al., 2009). Moreover, leakage through the BBB varies in different experimental stroke models (Klohs et al., 2009; Abulrob et al., 2011). Of note, post-stroke BBB leakage also varies in different areas of the brain, whereas the administration of the broadly used thrombolytic agent tissue plasminogen activator increases leakage (Jaffer et al., 2013), resulting in hemorrhagic transformation. Moreover, a recent evidence of the pivotal contribution of BBB leakage to ischemic stroke damage has been revealed by a study in which a vascular leakage blocker reduced stroke damage (Zhang et al., 2017).

### Effect of Stroke on Brain Iron

Regarding iron content of the brain following ischemic stroke, an increase in total iron was observed in the ischemic areas after permanent or transient ischemic stroke (Helal, 2008; Millerot-Serrurot et al., 2008; Tuo et al., 2017). In addition, increased TfR and iron levels were observed in susceptible hippocampal areas following transient global cerebral ischemia (Park et al., 2011), or increased extravasation of iron-loaded HTf was observed following acute focal ischemic stroke (DeGregorio-Rocasolano et al., 2018). In fact, HTf from blood accumulates in the brain parenchyma of the rat ischemic brain hemisphere at 1 h after reperfusion (DeGregorio-Rocasolano et al., 2018). Subsequently, iron accumulates into the endothelial cells of brain capillaries near ischemic areas (Desestret et al., 2009), whereas increased levels of Tf were observed 24 h following a transient experimental stroke at the ischemic hemisphere (Lo et al., 2007). In fact, this Tf seems to accumulate in neurons since Tf immunoreactivity was found located at the neuronal cytoplasm 24 h after stroke onset (DeGregorio-Rocasolano et al., 2018).

In the ICH stroke type, the systemic iron pools play a direct role in brain damage since the rupture of the microvascular wall and concomitant blood release results in neurons, astrocytes, and other cells around the hemorrhagic area to become immediately exposed to blood-derived iron, either free or bound to Tf (Figure 1, 2). Moreover, the brain is exposed to a bulk amount of erythrocytes that undergo lysis and subsequent spill of hemoglobin (Hb). Erythrocyte lysis occurs within minutes and continues for days after the formation of brain hematoma, leading to the release of red blood cell-derived products. Cells of the brain are then sequentially exposed to aberrantly high extracellular levels of Hb, the oxidized Hb form methemoglobin, and free heme or the iron ferric heme form hemin. The main mechanisms of neurodegeneration in hemorrhagic stroke are iron- and heme-induced ROS production, amplification of an inflammatory response, direct toxic effects of iron and heme, and glutamate-induced excitotoxicity (Righy et al., 2016).

## Brain Regulation of Iron Metabolism During Stroke

### Endogenous Mechanisms Involved in the Protection of Brain Cells From Excess Iron Following AIS

Increased brain Tf or TfR1 levels (Ding et al., 2011; DeGregorio-Rocasolano et al., 2018) were observed in lethal ischemia. Consensus exists about the protective role of increased FT expression during experimental stroke, and the same applies to hepcidin in AIS; however, the role of blunting the ischemia-induced upregulation of Tf and TfR remains controversial. Reductions of Tf and TfR were considered to be associated with neuroprotection in one specific stroke model (Lo et al., 2007). However, other neuroprotective conditions were not associated with Tf and/or TfR reductions (He et al., 2012).

Hepcidin seems to protect from neurodegeneration in AIS. It is an iron-inducible peptide hormone that is considered to be the main regulator of iron homeostasis. Hepcidin is produced as a propeptide mainly in the liver and then secreted to the blood where it interacts with the only known iron exporter FPN located at the cell membrane. Hepcidin acts as a negative regulator of cellular iron release by binding FPN and causing its internalization and degradation; this inhibits FPN-mediated iron egression from enterocytes, macrophages, hepatocytes and, as recently found, some other cell types (e.g., endothelial cells or neurons). The inhibition of iron egression, mainly from macrophages and hepatocytes, prevents the presence of NTBI in the blood as well as reduces the TSAT in the blood. In line with its main function, hepcidin deficiency leads to iron overload (Nicolas et al., 2001), and hepcidin overexpression causes severe iron deficiency (Nicolas et al., 2002a). Hepcidin has a rapid and direct impact on circulating iron pools. In fact, even a single intraperitoneal administration of hepcidin reduces the TSAT in blood to less than half the normal levels for 6-12 h (Sillerova et al., 2012). Both blood hepcidin and HTf/apotransferrin ratio might be considered as the master regulatory factors of systemic iron metabolism, since hepcidin senses iron by sensing HTf levels in the circulation and iron-FT levels inside hepatocytes. Relevant to this review on iron and stroke, hepcidin expression is also responsive to hypoxia (Nicolas et al., 2002b; Sonnweber et al., 2014) mainly by the oxygen-regulated hypoxia-inducible factor. In general, hypoxia inhibits hepcidin expression to allow the mobilization of iron to sustain erythropoietic expansion. However, a recent study showed that the exposure of mice to an hypoxic environment induces biphasic modulation of hepcidin expression with a transient increase in liver expression within the first 6 h and a reduction after 15 h of hypoxia (Ravasi et al., 2018).

Among ischemic stroke patients, who have areas of the brain exposed to oxygen and glucose deprivation (OGD), blood hepcidin levels increased within the first 6 h from symptom onset compared to those in controls; serum hepcidin levels in ICH patients were similar to those of controls (unpublished results from our laboratory). This early increase in blood hepcidin during ischemic stroke is in agreement with the early increase reported in the hypoxic mice model (Ravasi et al., 2018) and with the two other reports on stroke patients (Petrova et al., 2015; Slomka et al., 2015) and might be a reflection of an increased expression and secretion of hepcidin from the ischemic brain cells to the extracellular medium, and a subsequent spillover out of the brain during stroke. Although hepcidin expression in the brain is thought to be low in basal conditions, hepcidin is now emerging as an important player in brain iron homeostasis (Vela, 2018). Hepcidin mRNA levels increased early in the brain in response to ischemia in a model of endothelin-induced vasoconstriction (Bickford et al., 2014), and hepcidin levels were significantly elevated in the ischemic side of the brain 24 h after occlusion in a stroke model due to middle cerebral artery occlusion; this ischemic side also showed increased TfR1 and FT levels and reduced FPN levels (Ding et al., 2011). In addition, knock-down of hepcidin slows the ischemic-mediated changes in brain FT and FPN, revealing an important contribution of this molecule to parenchyma iron regulation in cerebral ischemia. Further, normoxic iron-overloaded rats, hepcidin, either overexpressed in the brain or administrated to the brain parenchyma, reduced brain uptake of iron *in vivo* as well as reduced iron uptake in cultures of neurons or brain microvascular endothelial cells, owing to the dowregulation of iron-transport proteins (Du et al., 2015). In microglial cells, the bone morphogenetic protein antagonist noggin abrogated the OGD-induced changes in hepcidin, FPN, and FT levels, and this inhibition of bone morphogenetic protein/hepcidin axis *in vitro* shifted reactive microglia from an iron-storing to an iron-releasing phenotype after OGD plus reperfusion (Shin et al., 2018). Factors released from noggin- and OGD-exposed microglial culture medium were found to increase myelin production in oligodendrocyte cell cultures; since this effect is blunted by the iron chelator deferoxamine (DFO), iron released from the microglia is proposed to serve in the remyelination in the ischemic brain (Shin et al., 2018).

### Endogenous Mechanisms Involved in the Protection of Brain Cells From Excess Iron Following ICH

Similar to that observed in AIS, subarachnoid hemorrhage has been shown to increase hepcidin expression in neurons (Zhao et al., 2018) associated with a reduction of its downstream target FPN (Tan et al., 2016). However, in this hemorrhage model, hepcidin seems to be harmful since its administration potentiated and its siRNA decreased apoptosis and early brain damage (Tan et al., 2016). In agreement with a harmful effect of hepcidin on brain hemorrhage, higher blood hepcidin levels were observed 1-7 days following ICH in patients with poorer outcomes (Xiong et al., 2015). The rationale for this harmful effect of hepcidin following ICH might be an impaired clearance of iron from brain parenchyma, since increased hepcidin expression caused by inflammation was found to inhibit intracellular iron efflux from brain microvascular endothelial cells and further draining of iron into circulation, leading to the aggravation of oxidative brain injury (Xiong et al., 2016). Conversely, addition of hepcidin or adenovirus-driven overexpression of hepcidin has been shown to protect neurons from hemin-induced injury (Zhou et al., 2017); this protection was thought to be related to the effect of hepcidin reducing hemin-induced iron uptake and reduced expression of TfR1 and DMT1.

Moreover, importantly, stroke has an inflammatory component, and some inflammatory inducers or mediators (e.g., lipopolysaccharide or interleukin 6) have been found to increase the expression of hepcidin in astrocytes, microglia, or neurons (for a recent review see Vela, 2018), although the effect on neurons is usually weaker (Ma et al., 2018).

The brain has protective mechanisms that can be activated after ICH and erythrocyte lysis, the most important one being the Hb-scavenging molecule haptoglobin (Hp) and the heme-scavenging system hemopexin (Hpx) (Figure 2). Hb itself is avidly bound by Hp to form a complex that prevents the release of the heme group from Hb or methemoglobin. However, Hp cannot prevent the release of the heme group from the highest oxidative Hb form ferrylHb. In the brain, Hp is almost exclusively synthesized by oligodendrocytes, and mice overexpressing Hp were more resistant, whereas Hp-knockout mice were more susceptible, to ICH injury (Zhao et al., 2011). The complex Hp:Hb binds to the Hp-specific receptor CD163 that is present in macrophages, allowing them to remove Hb from the extracellular space. In addition, CD163 is upregulated in neurons after ICH (Liu et al., 2017). The role of Hp in neuroprotection was not devoid of controversy since Hp was found to exacerbate the vulnerability of CD163-expressing neurons to Hb, an effect that was reduced by iron chelators (Chen-Roetling and Regan, 2016). However, most Hb was not bound to Hp, indicating that the CD163–Hb–Hp system is saturated, and that the primary route for Hb clearance from the CNS occurs through the BBB (Galea et al., 2012; Righy et al., 2018). A good clearance is associated with neuroprotection since lower CSF Hp levels were found in ICH patients without clinical and/or radiological evidence of delayed cerebral ischemia (Galea et al., 2012). Hb accumulated in the brain parenchyma is converted to ferrylHb, which causes the upregulation of proinflammatory adhesion molecules and increases the recruitment of inflammatory neutrophils or macrophages (Jeney et al., 2013).

Iron–hemin uptake has been reported in neurons and astrocytes and is especially high in microglial cell cultures; neurons benefit from a high export of iron following the initial iron–hemin loading, and this export was exacerbated by treatment with DFO, a compound that attenuates hemin neurotoxicity (Chen-Roetling et al., 2014). As already mentioned, the brain produces the heme scavenger protein Hpx that protects from deleterious effects of heme. In this regard, deletion/knockout of the Hpx aggravates brain injury by ICH (Chen L. et al., 2011; Ma et al., 2016) and, although endogenous levels of Hpx are insufficient to counteract the massive heme overload following ICH, increasing brain Hpx levels has been found to improve the outcome after ICH (Leclerc et al., 2018). Conversely, Hpx has recently been reported to increase the neurotoxicity of Hb in the absence of Hp (Chen-Roetling et al., 2018). Therefore, although both Hp and Hpx might provide a robust line of defense during ICH, additional knowledge on the fine tuning of this system is still required to design new possible neuroprotective treatments for ICH. The low-density lipoprotein receptor-related protein CD91 is expressed in several cell types, including macrophages and neurons, and was identified as the Hpx–heme receptor, mediating Hpx–heme uptake by endocytosis (Hvidberg et al., 2005). Once inside the cell, the heme oxygenase (HO) system is responsible for cellular heme degradation. In neurons, HO generates biliverdin, which has been reported to scavenge free radicals. Astrocytes rapidly upregulate HO1 and FT and are resistant to heme-mediated injury, and astrocyte HO1 is known to play a robust neuroprotective role after hemorrhage (Chen-Roetling et al., 2017). A recent study indicated that Hpx activates endothelial progenitor cells and reduces synaptic damage in a rodent ischemic stroke model; these effects require HO1 activity (Yang et al., 2018).

Moreover, the presence of heme groups in the extracellular milieu activates the endothelial expression of adhesion molecules and promotes the activation of neutrophil extracellular traps through a mechanism dependent on ROS generation (Chen et al., 2014). Further, inflammatory response is a well-documented mechanism of brain damage after ICH (Wang and Doré, 2007), and the mechanisms underlying heme-induced inflammation have been reviewed elsewhere (Dutra and Bozza, 2014). Iron and heme associated with pro-inflammatory mediators in the CNS following hemorrhagic stroke and inflammatory profiles are associated with poorer prognosis. Similarly, iron-induced damage following ischemic stroke in iron-overloaded animals has also been related to increased inflammatory responses associated with increased serum levels of interleukin 6 and TNF-alpha (Castellanos et al., 2002).

## The Ferroptotic Component of Stroke-Induced Neurodegeneration

### The Concept of Ferroptosis: Molecular Players Involved in Ferroptosis in the Neurons

The concept of ferroptosis was introduced as a form of cell death, which is morphologically, biochemically, and genetically distinct from apoptosis, necrosis, and autophagy; it is associated with excessive iron-mediated accumulation of lipid ROS in cancer cells as well as in hippocampal slice cultures exposed to glutamate excitotoxicity (Dixon et al., 2012). As excitotoxicity is the main mechanism involved in neuronal death in ischemic stroke, the involvement of ferroptosis in ischemic stroke could be suspected. In addition, the role of high systemic iron exacerbating stroke-induced damage, concomitant with increased brain iron after ischemic stroke onset (reviewed in the previous section), further suggested a possible ferroptosis component in stroke; in the recent past, mounting evidence indicated that ferroptosis plays such a pivotal role in stroke-induced neurodegeneration in both ischemic and hemorrhagic stroke subtypes. Although the concept is still evolving, ferroptosis is now considered a unique form of regulated cell death characterized by cytosolic accumulation of iron and executed by the massive peroxidation of PUFA in the plasma membrane and associated with reduced glutathione (GSH). Compounds that induce only either cytosolic or mitochondrial ROS production are not considered as ferroptosis inducers. A recent study proposed that both Fenton reactions, in which Fe^2+^ converts to Fe^3+^ producing ROS, and enzymatic lipoxygenase-mediated reactions in cellular membranes contribute to ferroptosis (Feng and Stockwell, 2018). Some authors considered that the ferroptosis concept and the previously described oxidative oxytosis concept have many common features to be regarded as independent processes (Lewerenz et al., 2018). In fact, the widely used oxidative stress stimulus *tert*-butylhydroperoxide was found to induce neuronal cell death that was blocked by ferroptosis inhibitors, thereby implying a crosstalk between the initial oxidative damage and the occurrence of ferroptosis (Wu et al., 2018). However, ferroptosis is considered a major driver of cell death in many neurodegenerative and neurological diseases (Lewerenz et al., 2018; Morris et al., 2018).

Ferroptosis can be triggered by the inhibitors of the lipid repair enzyme glutathione peroxidase 4 (GPX4), such as the RAS-selective lethal 3 compound, or depletion of the antioxidant GSH by starving the cells of GSH precursors [inhibiting the cystine/glutamate antiporter, i.e., system $X_{c}^{-}$ ($X_{c}^{-}$) with erastin, sulfasalazine, sorafenib, or, alternatively, challenging the exchange capacity of $X_{c}^{-}$ with high extracellular glutamate]. Extracellular Tf in its iron-loaded HTf form is considered as an inducer of ferroptosis as well, and inhibiting TfR expression prevents ferroptosis (Gao et al., 2015).

At present, the existence of $X_{c}^{-}$ in neurons is still controversial, with one report showing expression lack of $X_{c}^{-}$ in neurons (Ottestad-Hansen et al., 2018), whereas another showing neurons expressing significant $X_{c}^{-}$ levels (Soria et al., 2016). However, neurons in culture overexpress $X_{c}^{-}$ shortly after being exposed to OGD (Soria et al., 2014). The physiological role of $X_{c}^{-}$ as a cystine importer is linked to its antiporter glutamate export and requires that extracellular levels of glutamate and other excitatory amino acids are maintained low by the action of excitatory amino acid transporters (EAATs). Thus, one of these glutamate transporters, the sodium-dependent EAAT3, might be selectively expressed in neurons and could also be an unidirectional cysteine import channel, with extracellular glutamate inhibiting its cysteine import capacity (Watts et al., 2014). EAAT3 might be as important as $X_{c}^{-}$ in providing GSH precursors to neurons, considering that mice lacking EAAT3 have lower GSH levels and age prematurely (Aoyama et al., 2006). Notably, low GSH levels might also inhibit GPX4 activity since GPX4 uses GSH as a cofactor to catalyze the reduction of lipid peroxides and protect cells membranes against peroxidation (see Latunde-Dada, 2017).

Conversely, ferroptosis can be prevented by (1) iron-binding compounds such as DFO and ciclopirox; (2) small molecules, free radical scavengers, or lipophilic antioxidants such as ferrostatin-1, α-tocopherol, butylated hydroxytoluene, or liproxstatin-1; (3) treatments with molecules such as deuterated PUFA, inhibitors of acyl-CoA synthetase long-chain family member 4, lipoxygenase inhibitors, glutaminolysis inhibitors, or cycloheximide; or (4) boosting cystine and/or cysteine import by reducing extracellular glutamate.

Glutamate is the most abundant excitatory neurotransmitters in the vertebrate nervous system, with 90% of the synaptic neurotransmission considered glutamatergic (Hofmeijer and Van Putten, 2012; Mayor and Tymianski, 2018). Under physiological conditions, extracellular glutamate levels are low, and glutamate exerts its physiological role mainly at the neuronal synapse. The ability of astrocytes near glutamatergic neurons to rapidly clear synaptic glutamate after its physiological action is critical in the functioning of synapses and neuronal circuits, and the initial increase of extracellular glutamate is inhibited mainly by glial EAAT1 and 2. Neuronal glutamate transporters, mainly EAAT3, also play a role in clearing extracellular glutamate excess. However, ischemia leads to energy failure and subsequent electrochemical gradient impairment, causing EAATs to act in a reversed manner, thereby extruding non-vesicular intracellular glutamate and resulting in large excess of extracellular glutamate and impairment of EAAT3 as a unidirectional cysteine import channel.

The impact of excess extracellular glutamate on neuronal survival is more than that on the cystine/glutamate exchanger $X_{c}^{-}$. In fact, most of the effect is produced by glutamate binding to specific glutamate receptors present in cell membranes of several cell types and, especially in neurons, *N*-methyl-D-aspartate (NMDA) subtype of glutamate receptors (NMDARs) (Guirao et al., 2017) that are coupled to an intracellular downstream complex of signaling effectors, which are known to initiate excitotoxic neuronal death following ischemia.

Although both glutamate and iron are essential for neuronal survival, excess glutamate can be toxic, and excess free iron through the Fenton reaction is a potentially toxic substance that can catalyze the production of extremely damaging ROS. During stroke, the impairment of BBB function allows increased access to brain of iron-carrying substances and, preceding neurodegeneration, iron accumulates in the ischemic areas (Helal, 2008); therefore, not surprisingly, iron overload conditions are associated with larger areas of brain damage in animals exposed to experimental ischemic stroke (Castellanos et al., 2002; Gamez et al., 2003; Mehta et al., 2004; García-Yébenes et al., 2012; DeGregorio-Rocasolano et al., 2018). Following experimental stroke or NMDAR overactivation, prevention of NMDA-induced neuronal iron uptake was found to be neuroprotective (DeGregorio-Rocasolano et al., 2018). Therefore, the crosstalk between neuronal homeostasis of iron and glutamatergic signaling is increasingly being supported by evidence and should not be disregarded as a therapeutic intervention target.

### Glutamate-Induced Iron Uptake in Neurons and Ferroptosis

Cheah et al. (2006) from Dr. Snyder’s laboratory published a study before the concept of ferroptosis was introduced and showed that the overactivation of NMDAR in neurons induces, in addition to the well-known NMDAR-mediated calcium influx, an NMDAR-mediated iron uptake. They also identified a signaling cascade whereby NMDAR regulated neuronal iron homeostasis and excitotoxicity through an NMDAR/nitric oxide/dexamethasone-induced Ras protein 1 (Dexras1) pathway that increased both non-Tf and Tf-mediated iron uptake from the extracellular medium through the neuronal plasma membrane (Cheah et al., 2006). A similar increase of iron uptake in hippocampal neurons was observed in a subsequent study in which either synaptic activity or NMDAR activity was boosted (Pelizzoni et al., 2011). Moreover, either ischemia or NMDA overactivation increased the uptake of HTf, resulting in an increase of cytosolic redox-active iron in neurons, whereas blockade of TfR1 reduced iron dyshomeostasis and NMDA-induced excitotoxicity (DeGregorio-Rocasolano et al., 2018). The original work from Snyder’s laboratory hypothesized that iron in its free form gains access to the neuronal cytosol through DMT1 present in the neuronal membrane. However, this would not explain the uptake of Tf-bound iron since the DMT1 isoform endogenously overexpressed in primary cultures of hippocampal neurons exposed to NMDA is DMT1-1B/IRE(+) (Haeger et al., 2010) and is found in endosomes/lysosomes in the cytoplasm (Pelizzoni et al., 2012), thereby not supporting a direct role of DMT1 in iron entry through the neuronal membrane. Following the above rationale of crossroads between excitotoxicity and ferroptosis, several *in vitro* and *in vivo* studies showed that neurons survive otherwise lethal NMDA or glutamate concentrations when treated with iron chelators such as DFO or deferasirox (Yu et al., 2009; Tian et al., 2017; Sakamoto et al., 2018). Moreover, a study showed that the deletion of Dexras1 reduced NMDA-induced iron uptake and NMDA toxicity in cortical neurons and retinal ganglion cells (Chen et al., 2013). Moreover, Dexras1-KO mice showed increased expression of the NR2A subunit of the NMDAR (Carlson et al., 2016), a subunit that is preferentially located at synapses and related to NMDAR pro-survival signaling; this strongly suggests the existence of a functional crosstalk between the NMDA-induced iron intracellular signaling and the physiological NMDAR function. Interestingly, intracellular iron released from lysosomes has been shown to modulate NMDAR synaptic excitability via Dexras1 (White et al., 2016), and iron is needed post-synaptically to activate Ca^2+^-dependent pathways downstream NMDAR (Muñoz et al., 2011). Further, glutamate-induced neuronal excitotoxicity was described as ferroptosis (Dixon et al., 2012).

In the widely reported scenario of elevated extracellular glutamate during ischemic stroke, NMDAR-induced iron import was upregulated in neurons associated with increased ROS production (Cheah et al., 2006), and system $X_{c}^{-}$ and EAAT3 cystine/cysteine import are impaired by high extracellular glutamate (Conrad and Sato, 2012), thus compromising neuronal iron homeostasis and GSH-mediated free radical detoxification, eventually leading to an excess lipid oxidative stress that causes ferroptotic damage. High GSH stores are pivotal for cell survival since GSH is required for GPX4 activity and is also thought to be the only molecular species having a significant role as a cytosolic ligand for Fe^2+^ (Philpott and Ryu, 2014). Even when ischemia is resolved in the brain owing to a successful reperfusion, other mechanisms involved in ferroptotic damage might become dangerous. In this regard, a recent study on Sertoli cells revealed that GPX4 is inactivated via GSH depletion following I/R injury (Li et al., 2018), and several studies showed that GPX4 gene inactivation causes hippocampal neurodegeneration (Seiler et al., 2008; Hambright et al., 2017) and a remarkable degeneration of motor neurons (Chen et al., 2015).

### Ferroptosis in AIS and ICH Stroke Subtypes: Effect of Ferroptosis Inhibitors and Iron Chelators

In addition to a pivotal role of ferroptosis in I/R-induced damage in several peripheral organs (Friedmann Angeli et al., 2014; Linkermann et al., 2014; Gao et al., 2015), a role of ferroptosis has been specifically identified in I/R-induced brain damage in transient ischemia, as brain damage was substantially reduced by the ferroptosis inhibitor liproxstatin-1 in an AIS model (Tuo et al., 2017). These authors also showed that a reduction of tau levels, a protein that facilitates iron export by trafficking APP to membranes to stabilize FPN, was found to precede a pro-ferroptotic iron accumulation in the brain following I/R (Tuo et al., 2017). APP mRNA possesses a functional IRE with sequence homology to that of FT, and its translation is responsive to free iron levels in the cytosol (Duce et al., 2010). In addition, APP has been reported to interact with FPN to promote iron release in several brain cell types (Duce et al., 2010; McCarthy et al., 2014). APP overexpression reduces free iron content in neuroblastoma cells (Wan et al., 2012), and either preventing APP transport to the cell surface or knocking down of APP results in iron accumulation in primary cortical neurons in culture (Lei et al., 2012). The complete role of APP in ferroptosis and neurodegeneration in AIS requires additional studies. However, further evidence supporting the involvement of ferroptosis mechanisms in AIS brain damage is provided by the overexpression of lipoxygenases following brain ischemia and the protection afforded by treatment with lipoxygenase inhibitors (Cui et al., 2010; Karatas et al., 2018). Further, a recent study showed that *N*-acetylcysteine (NAC), an FDA-approved cysteine prodrug, prevents hemin-induced ferroptosis by neutralizing toxic lipids generated by arachidonate-dependent activity of 5-lipoxygenases (ALOX5) (Karuppagounder et al., 2018). They reported that ALOX5 inhibitors such as zileuton and molecules such as NAC that inhibit toxic metabolic products of ALOX5 or prevent ALOX5 recruitment to its site of action, protect against hemin or ICH-induced ferroptosis (Karuppagounder et al., 2018). Notably, NAC has been previously reported to prevent neuronal damage induced by AIS (Khan et al., 2004; Turkmen et al., 2016).

Further, the role of free iron in ferroptosis and neurodegeneration is further supported by the protective effect of treatments with exogenous iron chelators, e.g., DFO or 2,2′-dipyridyl, in animal models of stroke. After permanent ischemic stroke (Millerot-Serrurot et al., 2008), the increase in free iron, the iron form prone to induce lipid peroxidation and susceptible to be chelated, precedes the increase in total iron. In experimental models of transient ischemic stroke, DFO administered intramuscularly (Xing et al., 2009) or intranasally (Hanson et al., 2009) after the induction of ischemia significantly decreased infarct volume. Another iron chelator, the aforementioned 2,2’-dipyridyl was found to be cytoprotective after permanent arterial occlusion in rats; this was associated with reduced infarct size and preservation of GSH levels (Demougeot et al., 2004) or reduction of the apoptotic component and prevention of the enlargement of the lesion (Van Hoecke et al., 2005).

The role of iron in ICH neurodegeneration has been known for long. Recently, neuronal death following ICH was found to have features similar to those of ferroptosis, since chemical inhibitors of ferroptosis protected against Hb- and hemin-induced neurotoxicity (Zille et al., 2017). The nonheme iron present in brain parenchyma after ICH is a source of redox-active iron. The marked increase in brain nonheme iron was not cleared for weeks following the ICH event, and brain Tf, TfR, and FT levels were also increased 72 h after ICH, thereby making FT upregulation long-lasting (Wu et al., 2003). Iron chelators such as DFO reduce hemin- and iron-mediated neurotoxicity, perihematoma edema, and neuronal damage, leading to good neurologic outcomes after ICH (Nakamura et al., 2004; Hatakeyama et al., 2013). Other recent studies indicated that the specific inhibitor of ferroptosis ferrostatin-1 reduces neuron degeneration and improves neurological deficit in experimental ICH models (Li Q. et al., 2017; Zhang et al., 2018), and GPX4 levels were found to be reduced after ICH, whereas pharmacological inhibition of GPX or genetic knockdown exacerbated brain injury after ICH (Zhang et al., 2018). Moreover, inhibitors of ALOX5 or inhibition of the ALOX5 metabolic products protected against hemin- or ICH-induced ferroptosis (Karuppagounder et al., 2018).

Moreover, DFO significantly reduced hemorrhagic transformation after focal I/R (Xing et al., 2009; Li Y. et al., 2017). Some studies have shown that the effect of DFO in preventing ischemic neuronal death is related to the DFO-induced expression of hypoxia-inducible factor 1 and its downstream targets genes rather than with its iron-chelating properties or, recently, with microglial/macrophage HO1 expression in ICH (LeBlanc et al., 2016). A systematic review and meta-analysis in experimental models of ICH showed that DFO was neuroprotective, e.g., reduction of brain edema and improvement of neurobehavioral outcomes, especially when it was administered 2-4 h after ICH induction (Cui et al., 2015). In clinical studies, conclusive evidence of the benefit of DFO treatment in ICH (Zeng et al., 2018) or AIS patients is still lacking; some clinical trials of DFO in ICH and AIS are currently ongoing.

The rationale for iron chelation as a neuroprotectant relies primarily on the idea that chelation removes free iron present in the blood and enhances iron elimination in the urine. After gaining access to other body compartments, DFO could also act by chelating free extracellular and cytosolic iron, whereas its effect on blood TSAT or blood total iron levels is modest (Tauchenová et al., 2016). In fact, DFO was shown to have an extremely slow removal rate of iron from Tf (Abergel and Raymond, 2008). This, together with the fact that DFO half-life in the plasma is extremely short (20 min), poses serious doubts about the effectiveness of the treatment with intravenous DFO unless administered with continuous infusion. Recently, another iron-regulator, apotransferrin, administered after stroke AIS onset, was found to be neuroprotective because of the systemic reduction of blood TSAT and a reduction of NMDA ischemia-induced neuronal iron import (DeGregorio-Rocasolano et al., 2018).

## Conclusion

Increasing number of studies show that ferroptosis is involved in several neurodegenerative diseases. This review summarizes the knowledge available thus far regarding the role of iron and ferroptosis in the execution of neuronal cell death during AIS and ICH. In AIS-induced cell death, the role of systemic iron, Tf, TSAT, and hepcidin on excitotoxic damage, infarct size, and neurological outcome in rodent models of stroke might have important clinical implications. Following the AIS event, high levels of extracellular glutamate and impairment of the BBB allow the NMDAR-mediated influx of iron, either bound or not to Tf, into neurons. In addition, high extracellular glutamate prevents the neuronal production of the antioxidant GSH or the function of GPX4, thereby promoting a form of cell death in which glutamate receptor overactivation boosts neuronal iron uptake, which in turn promotes an overwhelming production of membrane peroxides. The role of iron in ICH neurodegeneration has been known for long, since blood spillover results in iron-Hb and iron–heme complexes at the extracellular milieu of brain parenchymal cells, thereby contributing to iron-induced lipid peroxidation and neuronal death. The review documents the beneficial effects of iron chelators such as DFO, physiological molecules with neuroprotective potential, and specific inhibitors of ferroptosis liproxstatin-1 or ferrostatin-1 in reducing neuron degeneration and improving neurologic deficits induced by ischemic and hemorrhagic stroke subtypes.

## Author Contributions

The three authors have contributed in a similar way to the writing of the article.

## Conflict of Interest Statement

The authors declare that the research was conducted in the absence of any commercial or financial relationships that could be construed as a potential conflict of interest.

## Abbreviations

AIS: acute ischemic stroke
ALOX5: arachidonic acid lipoxygenase 5
APP: amyloid precursor protein
BBB: brain–blood barrier
CSF: cerebrospinal fluid
Dexras1: dexamethasone-induced Ras protein 1
DFO: deferoxamine
DMT1: divalent metal transporter 1
EAATs: excitatory amino acid transporters
FPN: ferroportin
FT: ferritin
FTH: ferritin heavy chain
GAPDH: glyceraldehyde-3-phosphate dehydrogenase
GPX4: glutathione peroxidase 4
GSH: glutathione
Hb: hemoglobin
HO: heme oxygenase
Hp: haptoglobin
Hpx: hemopexin
HTf: holotransferrin
ICH: intracerebral hemorrhage
I/R: ischemia/reperfusion
IREs: iron regulatory elements
IRPs: iron regulatory proteins
NAC: *N*-acetylcysteine
NMDA: *N*-methyl-D-aspartate
NTBI: non-transferrin-bound iron
OGD: oxygen and glucose deprivation
PUFA: polyunsaturated fatty acids
ROS: reactive oxygen species
TIM: T-cell immunoglobulin and mucin domain-containing receptor
Tf: transferrin
TfR1: transferrin receptor 1
TfR2: transferrin receptor 2
TSAT: saturation of transferrin with iron
UTRs: untranslated regions
$X_{c}^{-}$: cystine/glutamate exchanger or antiporter, i.e., SLC7A11/xCT
